# Mechanism of Heshouwuyin inhibiting the Cyt c/Apaf-1/Caspase-9/Caspase-3 pathway in spermatogenic cell apoptosis

**DOI:** 10.1186/s12906-020-02904-9

**Published:** 2020-06-11

**Authors:** Hongjie Wang, Juan Zhu, Liping Jiang, Boying Shan, Peihan Xiao, Jiayi Ai, Na Li, Feng Qi, Siyun Niu

**Affiliations:** 1 School of Medicine, Hebei University, Baoding, 071002 Hebei Province China; 2 Affiliated Hospital of Hebei University, Baoding, 071002 Hebei Province China; 3 Nanbao Development Zone Hospital, Tangshan, 063305 Hebei Province China; 4 Baoding No.1 Hospital, Baoding, 071000 Hebei Province China

**Keywords:** Apoptosis, Heshouwuyin, Spermatogenic cells, Apaf-1

## Abstract

**Background:**

The Chinese herbal compound Heshouwuyin has been shown to downregulate the apoptotic rate of testicular tissue cells in Wistar naturally aging rats, and this effect might be related to the mitochondrial pathway [15]. Apoptotic protease activating factor-1 (Apaf-1) is a major component of the apoptotic complex, which is a key element of the mitochondrial endogenous apoptotic pathway [13]. To further clarify the mechanism of Heshouwuyin in the mitochondrial apoptotic pathway, this study used Apaf-1 as a target to explore the mechanism by which Heshouwuyin inhibits the Apaf-1 pathway of spermatogenic cell apoptosis.

**Methods:**

In this study, an aging model of rat spermatogenic cells was established using free radical oxidative damage. Flow cytometry was used to detect the apoptosis rate of germ cells and the inhibitory effect of Heshouwuyin. Apaf-1 was specifically knocked down by siRNA interference technology, and mitochondrial membrane potential was measured. qRT-PCR, Western blotting and immunofluorescence analyses were used to detect the expression of the key genes Cyt c, Caspase-9 and Caspase-3 in the mitochondrial apoptotic pathway of spermatogenic cells.

**Results:**

Heshouwuyin reduced the mRNA and protein expression levels of Cyt c, Caspase-9 and Caspase-3 in senescent spermatogenic cells. In these cells, the mRNA and protein expression levels of Cyt c did not change significantly after specific knockdown of Apaf-1, and the mRNA and protein expression levels of Caspase-9 and Caspase-3 decreased significantly. This finding indicated that knockdown of Apaf-1 could decrease the mRNA and protein expression levels of the downstream pro-apoptotic genes Caspase-9 and Caspase-3. Although Cyt c was an upstream gene of Apaf-1, knockdown of Apaf-1 had no significant effect on Cyt c expression.

**Conclusion:**

The inhibition of spermatogenic cell apoptosis by Heshouwuyin was closely related to the Cyt c/Apaf-1/Caspase-9/Caspase-3 pathway. The inhibition of apoptosis by Heshouwuyin not only involved the Apaf-1 pathway, but other signaling pathways.

## Background

Apoptosis is a genetic control process of selective cell removal and is closely related to individual growth, development and aging [1]. During spermatogenesis, any pathological or environmental factors may disrupt the balance between germ cell survival and apoptosis, leading to azoospermia, weak sperm disease, etc. [2]. Traditional Chinese medicine compounds have significant clinical effects that delay aging and treat infertility and have attracted widespread attention [3, 4]. The Chinese herbal compound Heshouwuyin is made from the classic clinically prescribed Heshouwu pill, which was created by the Chinese traditional medicine doctor Liu Hejian. Previous studies have shown that Heshouwuyin significantly improves serum testosterone levels in aging rats and has a regulatory effect on p53/pRb-related proteins in the testicular tissue cell senescence pathway [5]. Heshouwuyin enhanced the activity of testosterone synthase in Leydig cells, promoted testosterone secretion, and improved the sperm quality of aging rats, and the curative effect was significantly better than that of Heshouwu pill [6]. In this study, flow cytometry was used to detect the inhibitory effect of Heshouwuyin on the apoptosis of senescent sperm cells. The results showed that Heshouwuyin can inhibit the apoptotic rate of senescent spermatogenic cells, which was consistent with the results of previous animal experiments [7].

The endogenous pathway for apoptosis is the mitochondria-mediated caspase activation pathway [8–10]. Recent studies have shown that protein complexes regulate cells by controlling and mediating signalling pathways, which act as signalling devices [11–13]. Apaf-1 is a major component of the apoptotic complex and a key element of the mitochondrial endogenous apoptotic pathway. After induction of apoptosis, Cyt c enters the cytoplasm in the presence of ATP, which in turn activates Apaf-1 and induces conformational changes in its protein, recruiting and activating procaspase 9 to form the apoptotic complex [14]. Apaf-1 is a major component of apoptotic bodies. The formation of apoptotic bodies is an upstream event of the mitochondrial pathway and is capable of initiating Caspase-3, a downstream encoding a cell death inducer. SVT016426, an Apaf-1 inhibitor, affects the formation of apoptotic bodies and inhibits apoptosis and maintains cell function [15]. Therefore, Apaf-1 can be used as a drug target to reduce the activity of downstream genes of the Caspase family by regulating Apaf-1, thereby regulating cell death. Heshouwuyin downregulated the apoptosis rate in testis tissue from natural aging rats [16] and promoted cell proliferation, and this effect might be related to the mitochondrial pathway [7]; however, it is unknown if its effect was achieved by inhibiting spermatogenic cell apoptosis. What was the mechanism? Did Apaf-1 regulate the mitochondrial apoptotic pathway to inhibit spermatogenic cell apoptosis?

## Methods

### Animals

Thirty SPF male Wistar rats weighing 320~360 g (2 months old) were selected from the Experimental Animal Center of Hebei Medical University (Animal license No. 1510063). Rats were housed in clean cages at a constant temperature (25 °C) and photoperiod (12 h light, 12 h dark). All experimental procedures were conducted according to the guidelines of the Animal Care and Ethics Committee of Hebei University, China. Disposal of experimental animals was performed in accordance with the Guidance Suggestions for the Care and Use of Laboratory Animals, formulated by the Ministry of Science and Technology of China.

### Dose

The compound medicine composition of Heshouwuyin is as follows: *Polygonum multiflorum* Thunb, *Cistanche deserticola*Y.C.Ma, *Achyranthes bidentata* B1., *Poria cocos (Schw.)* Wolf, *Epimedium brevicornu* Maxim., and *Salvia miltiorrhiza* Bge. at a mass ratio of 3:2:3:2:5:3. Formula granules were selected from the formula granules produced by Guangdong Yifang Pharmaceutical Co., Ltd. The equivalent ratio of decoction pieces and granules was *P. multiflorum* Thunb (1:10), *C. deserticola* Y.C. Ma (1:10), *A. bidentata* B1. (1:5), *E. brevicornu* Maxim. (1:20), *S. miltiorrhiza* Bge. (1:10), and *P. cocos* (Schw.) Wolf (1:5). Guangdong Yifang Pharmaceutical Co., Ltd. undertook the formal identification of the plant material used in the study. The control numbers from the product inspection report for these plants are as follows: *Polygonum multiflorum* Thunb (171020C2004), *Cistanche deserticola**Y.C.Ma* (171226C2005), *Achyranthes bidentata B1.*(180306C2007), *Poria cocos (Schw.) Wolf* (171108C2015), *Epimedium brevicornu Maxim* (171119C2008), *Salvia miltiorrhiza Bge* (180103C2001).

Heshouwuyin contained 2.4 g/100 g of crude drug, which is equivalent to the dose for an adult human. The results of previous studies have shown that doubling the human dose to 4.8 g/100 g crude drug is optimal for administration to rats [17]. Based on the formulation of our pellets and that of the granules produced by Guangdong Yifang Pharmaceutical Co., Ltd., as well as the conversion factor for rats and humans, the dose administered to rats in our study was 0.56 g/100 g of body weight (obtained by dissolving 0.56 g of prepared Heshouwuyin in 0.8 ml of normal saline; the resulting concentration corresponded to 0.7 g/ml).

### Preparation of drug-containing serum

SPF male Wistar rats (2 months old), weighing 320~360 g, were gastrointestinally administered Heshouwuyin (prepared as described above) twice a day for 7 consecutive days to obtain Heshouwuyin-containing serum. On day 7, the rats were anaesthetized with sodium pentobarbital (50 mg/kg) 1 h after drug administration, and blood was aseptically withdrawn from the abdominal aorta. The serum was separated, inactivated at 56 °C for 30 min, filtered with 0.22 μm filters, aseptically aliquoted, and stored at − 80 °C. After the blood was taken from the abdominal aorta, the abdominal aorta was cut off, until death.

### Cell culture and identification

**Fig. 1:**
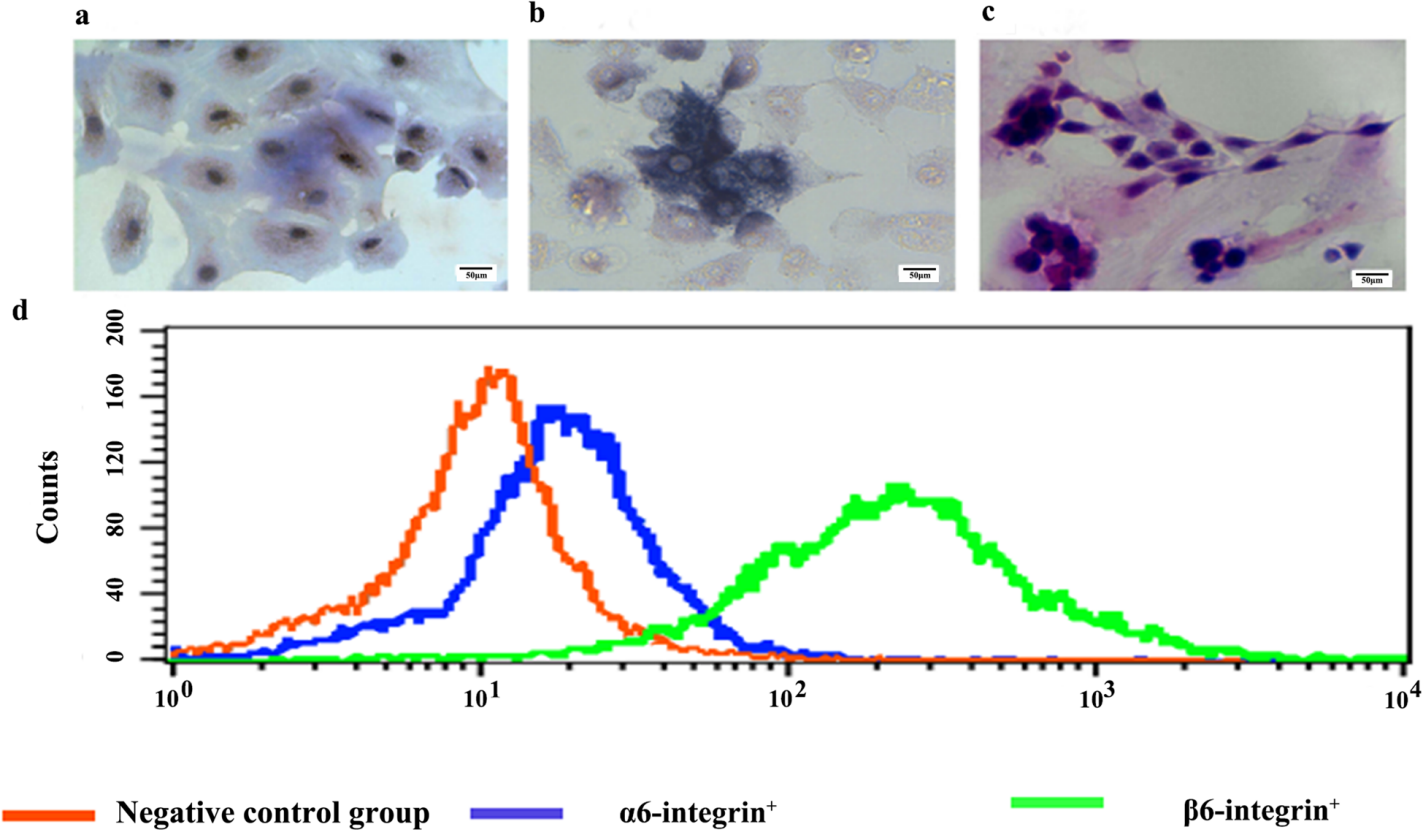
Identification of Sertoli cells, spermatogenic cells and SSCs (bar = 50 μm). **a**: Sudan IV staining of Sertoli cells; **b**: alkaline phosphatase staining of spermatogonia; **c**: c: H&E staining of spermatogenic cells; **d**: results of flow cytometry identification of SSCs

Sertoli cells were collected as follows: Cervical detachment on the 15th to 20th day after the birth of male rats [18], bilateral testes were taken, and the spermatic tubules were broken by digestion with type IV collagenase and trypsin and then cultured in DMEM/F12 containing 10% FBS. After being cultured at 35 °C, 5% CO_2_ for 4 h, the supernatant was transferred to an incubator (Leydig cells and fibroblasts were removed). After being cultured at 35 °C, 5% CO_2_ for 3 days, the cells were stained with Sudan IV. The cytoplasm showed enrichment for orange-red lipid droplets, which accumulated at the cytoplasmic poles or were dispersed around the nucleus (Fig. 1a). The cell purity was assessed to be more than 90%.

Spermatogonial stem cells (SSCs) were prepared as follows: SSCs were preliminarily isolated from testicular tissues by two-step enzyme digestion from testis tissues of 7- to 9-day-old Wistar rats [19], and the rats were executed by cervical detachment. After further purification by the differential adherence method, SSCs were cultured in DMEM/F12 containing 15% FBS at 35 °C, 5% CO_2_ for 3 h. After most Leydig cells and fibroblasts attached, the supernatant was transferred, and the cells were counted. Sertoli cells were inoculated at 3–5 × 10^5^ cells/ml and cultured. After the abovementioned Sertoli cells had been cultured for 5 days, the cells were collected by trypsin digestion, and the cell density was adjusted to 2–3 × 10^5^ cells/ml. After 48 h of inoculation, the SSCs were inoculated for 3 days, and the SSCs were identified by SSC-specific surface receptors (α6-integrin and β1-integrin) [20]. After 7 days of culture, spermatogenic cells were identified by alkaline phosphatase staining and haematoxylin and eosin (H&E) staining.

The staining results showed that the expression levels of the negative control, α6-integrin^+^ and β1-integrin^+^ cells were 2.56, 18.69, and 97.92%, respectively. Compared with that of the negative control cells, the fluorescence intensity of α6-integrin^+^ and β1-integrin^+^ cells was increased, and the peak values increased (Fig. 1d). SSCs were stained blue-black with alkaline phosphatase, and Sertoli cells did not take up any of the alkaline phosphatase stain; however, spermatocytes, spermatids, and the spermatozoa showed only low levels of staining (Fig. 1b). H&E staining indicated that spermatogenic cells had a round morphology, formed clusters and were stained purple, while supporting cells were stained lavender and had an irregular morphology (Fig. 1c).

### Establishment of the cell aging model

**Fig. 2:**
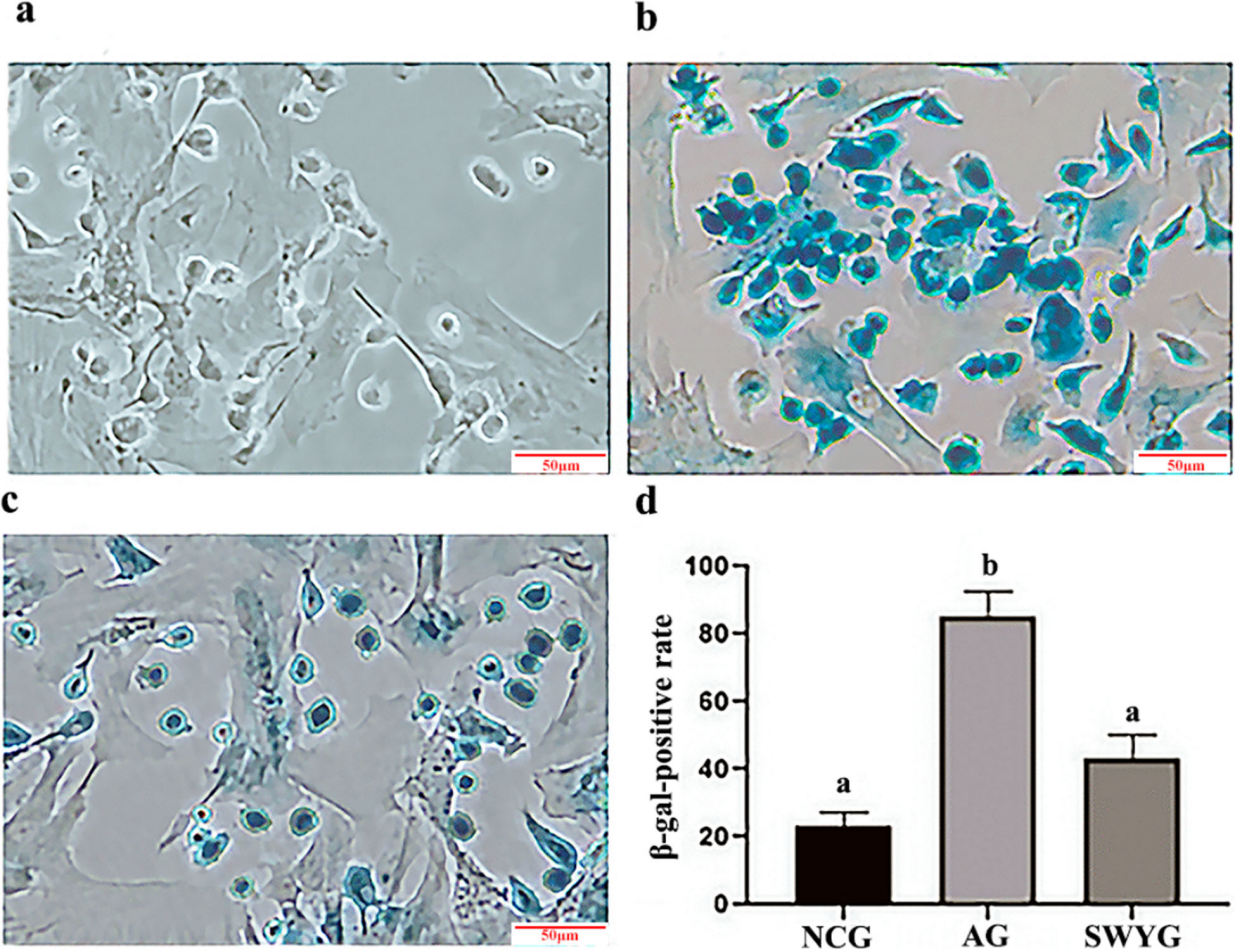
Identification of the cell senescence model by β-galactosidase staining (bar =50 μm) **a**: NCG; **b**: AG; **c**: SWYG; **d**: Statistical analysis of the β-gal positive rate in each group of cells. ^*a*^*p <* 0.05 vs. NCG; ^*b*^*p <* 0.05 vs. SWYG (*n* = 3)

The aging model of testicular spermatogenic cells was established by free radical oxidative damage, and the isolated and cultured SSCs were co-cultured with Sertoli cells in DMEM/F12 containing 15% FBS for 7 days followed by the addition of 50 μmol/l H_2_O_2_ and 100 μmol/l FeSO_4_, both in a volume of 2 μl. After 8 h of treatment, the medium was replaced with fresh medium, and the cells were cultured for an additional 72 h. The β-galactosidase staining method (Beyotime, China) was used to identify whether the cell senescence model was successfully established [21], and the rate of β-galactosidase-positive cells in spermatogenic cells was over 80% (Fig. 2).

### Did Heshouwuyin inhibit senescent spermatogenic cell apoptosis?

**Fig. 3:**
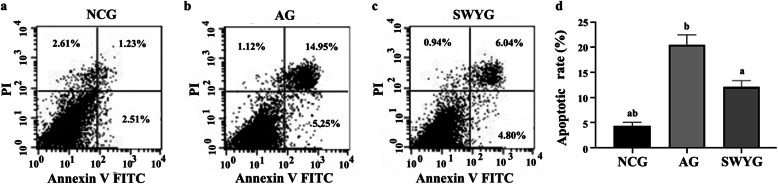
Effect of Heshouwuyin on apoptosis of spermatogenic cells. **a**: NCG; **b**: AG; **c**: SWYG. ^*a*^*p <* 0.05 vs. AG; ^*b*^*p <* 0.05 vs. SWYG (*n* = 3)

The flow cytometry results to detect apoptosis were as follows [22]: normal spermatogenic cells, senescent spermatogenic cells and senescent spermatogenic cells treated with Heshouwuyin were collected and centrifuged in a 15 ml centrifuge tube at room temperature at 1000 RPM for 5 min. The supernatant was carefully absorbed, and the cells were resuspended with a precooled PBS solution at room temperature at 1000 RPM for 5 min and washed twice. Then, 100 μl of pre-prepared binding buffer was added to the centrifuge tube, and the cell suspension density was adjusted to 1 × 10^6^ cells/ml. After blowing and mixing, the cells were screened, and the cell suspension was transferred to the flow tube. An annexin V-FITC&PI cell apoptosis detection kit (Sangon Biotech, China) was used to detect apoptosis. Annexin V-FITC and PI were added to each flow tube with 5 μl, respectively. The cells were incubated in the dark for 15 min at room temperature. Binding buffer (400 μl) was added to each tube, and the mixture was mixed and incubated. The results showed that the apoptosis rate of senescent spermatogenic cells was significantly higher than that of normal control cells, and Heshouwuyin inhibited the apoptotic rate of senescent spermatogenic cells in a manner that was statistically significant (Fig. 3).

The results indicated that Heshouwuyin could significantly reduce the apoptosis of senescent spermatogenic cells. We conducted the following experiments to determine if this effect was accomplished through the Apaf-1 pathway.

### Optimal time and optimal sequence screening of Apaf-1 gene siRNA interference

**Fig. 4:**
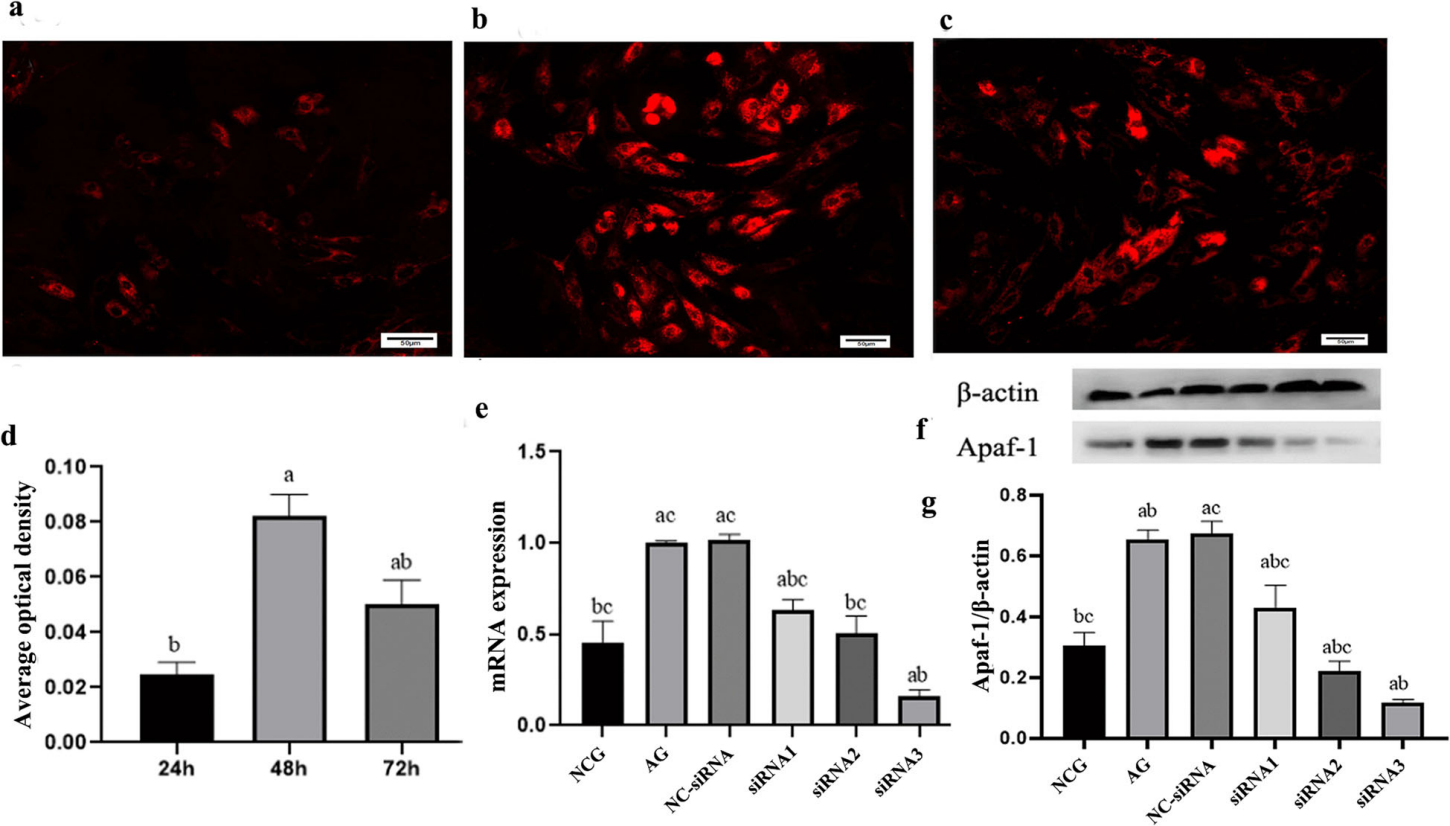
Screening for the optimal time and optimal sequence of siRNA-Apaf-1 (bar =50 μm) **a**, **b**, **c**: Transfection was performed at 24 h, 48 h, and 72 h; **d**: mean optical density statistics of Cy3 in spermatogenic cells transfected at 24 h, 48 h and 72 h (^*a*^*p <* 0.05 vs 24 h; ^*b*^*p <* 0.05 vs 48 h); **e**: Apaf-1 mRNA expression level of spermatogenic cells; **f**, **g**: Apaf-1 protein expression in spermatogenic cells (^*a*^*p <* 0.05 vs. NCG; ^*b*^*p <* 0.05 vs. NC-siRNA-transfected group; ^*c*^*p <* 0.05 vs. siRNA3-transfected group) (*n* = 3)

Immunofluorescence was used to detect Cy3 expression and to screen the optimal siRNA transfection time. Fluorescently labelled siRNAs were transfected into the spermatogenic cells for 24 h, 48 h and 72 h to detect the expression of Cy3 in the spermatogenic cells at each time point. The successfully transfected spermatogenic cells showed red fluorescence. The results showed that after 48 h of siRNA transfection, the fluorescence expression of Cy3 in spermatogenic cells was highest (Fig. 4a, b, c, d). Three different siRNA sequences were used to interfere with spermatogenic cells. The protein and mRNA expression levels of apaf-1 in spermatogenic cells were detected by Western blot and RT-qPCR analyses, and the optimal interfering sequences were selected (Table 1). The results showed that Apaf-1-siRNA could significantly inhibit the mRNA and protein expression levels of Apaf-1 in spermatogenic cells. siRNA3, which had the highest inhibition rate, was selected for subsequent experiments (Fig. 4e, f, g).

**Table 1:** Interference sequences of Apaf-1-siRNA

	Forward	Reverse
siRNA1	5‘ACAACUUCCUGGCCUAUCAdTdT 3′	3′dTdTUGUUGAAGGACCGGAUAGU5‘
siRNA2	5‘ACAGCCAUUUCCUAACAUUdTdT 3′	3‘dTdTUGUCGGUAAAGGAUUGUAA5‘
siRNA3	5‘GAGCCAUUAAGAUUAUAGAdTdT 3‘	3‘dTdTCUCGGUAAUUCUAAUAUCU5‘

### Cell grouping and treatment

According to the experimental purpose, the cells were divided into the normal control group, aging group, Heshouwuyin group, NC-siRNA-transfected group, Apaf-1-siRNA-transfected group and co-incubation group (Heshouwuyin + Apaf-1-siRNA).

**Fig. 5:**
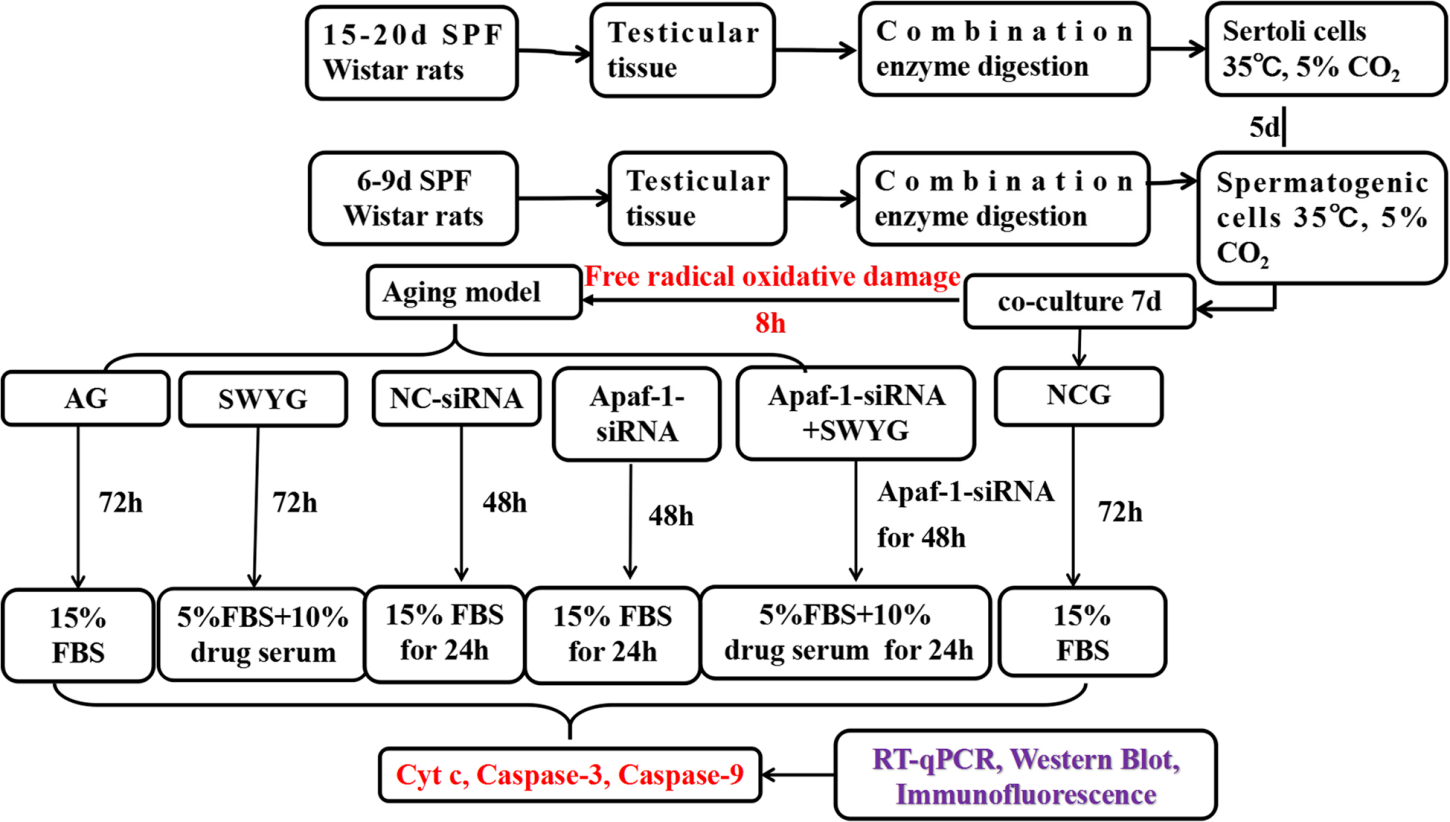
Schematic representation of cell groupings in this study

After 7 days of co-culture of spermatogenic cells and Sertoli cells in each group, the normal group was further cultured in DMEM/F12 containing 15% FBS for 72 h. All other groups were cultured in media with final concentrations of 50 μmol/l H_2_O_2_ and 100 μmol/l FeSO_4_ for 8 h, and then H_2_O_2_ and FeSO_4_ were removed. The aging group was further cultured in DMEM/F12 containing 15% FBS for 72 h. The Heshouwuyin group was cultured for 72 h by adding H_2_O_2_ and FeSO_4_, adding DMEM/F12 containing 10% drug-containing serum and 5% FBS. After removal of H_2_O_2_ and FeSO_4_, the corresponding NC-siRNA and Apaf-1-siRNA3 transfection complexes were added into the NC-siRNA-transfected group, the Apaf-1-siRNA3-transfected group and the co-incubation group for 48 h; the cells were then further cultured until 72 h in DMEM/F12 containing 15% FBS. H_2_O_2_, FeSO_4_, and 5% FBS containing 10% drug serum were added to the co-incubation group, which was further cultured for 72 h in DMEM/F12(Fig. 5).

### Determination of the mitochondrial membrane potential (MMP)

MMP was detected with a mitochondrial membrane potential kit (Beyotime,China). Fluorescence microscopy and a fluorescence microplate reader were used to observe the MMP [23, 24]. The experiment was repeated three times with three duplicate wells in each group.

### Immunofluorescence staining

The culture media were discarded from the 6-well plates, and the plates were washed with PBS. Spermatogenic cells were fixed with 4% paraformaldehyde for 30 min, then 0.5% Triton X-100 (Sigma, USA) was added for 20 min at room temperature, and the plates were washed with PBS. The spermatogenic cells were incubated with 10% goat serum for 20 min to block nonspecific binding sites. Cells were then incubated with a primary antibody working solution (1:200, Cyt c, Caspase-9, and Caspase-3, Abcam, USA) in an immunohistochemical wet box at 4 °C overnight. Then, spermatogenic cells were washed with PBS and incubated with a secondary antibody at 37 °C for 30 min in the dark. After washing the cells with PBS again, DAPI (Sigma, USA) was added for 30 min to stain the cell nuclei at room temperature in the dark; samples were then washed with PBS 3 times for 5 min each. An anti-fluorescence attenuation sealing tablet was added to seal the samples. Fluorescence microscopy was used to observe the experimental results. The entire experiment was repeated three times. Ten views were selected and observed from each of the 5 samples from every group. Finally, ImageJ was used to calculate the average optical density and conduct statistical analysis.

### Western blot analyses

Spermatogenic cells were lysed in RIPA buffer (PMSF:RIPA =1:99, Beyotime Inst. Biotech, China). The Pierce BSA Protein Assay Kit was used to measure the protein concentration. The concentrated glue was prepared and separated with the corresponding buffer concentration. After the protein sample (60 μg) was added, the target proteins were isolated by SDS-polyacrylamide gel electrophoresis (SDS-PAGE) and transferred to polyvinylidene difluoride (PVDF) membranes (Millipore, Atlanta, GA, USA). The PVDF membranes were blotted with 5% skim milk powder for 1.5 h at 37 °C. Then, the samples were washed with PBS-T three times for 5 min each and incubated with primary antibodies diluted with PBS-T (anti-β-actin, Proteintech, 1:5000; anti-Apaf-1, Cell Signaling Technology, anti-1:1000; anti-Caspase-9, Abways, 1:1000; anti-Caspase-3, Cell Signaling Technology, 1:2000; and anti-Cyt c, Abcam, 1:1000) at 4 °C overnight.

The samples were washed with PBS-T six times for 5 min each time on a shaker. The HRP-conjugated secondary antibody corresponding to the primary antibody (1:5000, Abways, Shanghai) was added and incubated with the samples for 1.5 h at 37 °C. The samples were washed with PBS-T six times for 5 min each time; then, the PVDF film was completely covered with ECL solution (liquid A:liquid B = 1:1). An enhanced chemiluminescence (ECL) system (Pierce) was used to develop the signals to detect the target proteins. The experiment was repeated three times. ImageJ was used to process the obtained images and to record the ID value (grey value) of each protein, and β-actin was used as an internal reference. The relative expression amount of target protein = the ID value of target protein/the ID value of β-actin.

### RNA isolation and qRT-PCR

qRT-PCR was used to detect the mRNA expression level of the mitochondrial apoptosis signals Cyt c/Apaf-1/Caspase-9/Caspase-3 in testicular spermatogenic cells. Total RNA from each group of spermatogenic cells was extracted by TRIzol (Aidlab Biotechnologies Co., Ltd., Beijing), and the concentration was determined. After the genomic DNA was removed with a reverse transcription kit (TaKaRa, Japan), 1 μg of total RNA was reverse transcribed into cDNA. In each group, 2 μl of cDNA was used as the amplification template, SYBR was used as a fluorescent dye and β-actin was used as an internal control. The primers Apaf-1, Cyt c, Caspase-9 and Caspase-3 were used as the upstream and downstream primers (Table 2). The reagents were added in order, mixed well, and amplified with a 7300 System Software PCR instrument (TaKaRa, Japan). All of the samples to be tested were replicated in three adjacent wells to reduce the operating error. The experiment was repeated three times, and the experimental results were analysed by the 2^-△△ct^ method.

**Table 2:** Primers for quantitative real-time PCR (qRT-PCR) used in this study

Gene	Primer sequences	Product size (bp)
Apaf-1	F:CGGCCCTGCGCATCTGATTCAT	288
	R:GGGCGAACGACTAAGCGGGACAG	
Cyt c	F:GCTAAACACCAGGACGGAACT	293
	R:CCACTCCCAATCAGGCATGAAC	
Caspase-9	F:CTGAGCCAGATGCTGTCCCATA	176
	R:GACACCATCCAAGGTCTCGATGTA	
Caspase-3	F:GACTGCGGTATTGAGACAGA	209
	R:CGAGTGAGGATGTGCATGAA	
β-actin	F:CCCATCTATGAGGGTTACGC	150
	R:TTTAATGTCACGCACGATTTC	

### Statistical analysis

The experimental data were analysed by SPSS 19.0 software and expressed as the mean ± standard deviation (SD); a difference was considered statistically significant at *p <* 0.05. First, a normality test of the experimental data was carried out. If the data followed a normal distribution, the mean was compared by single-factor analysis of variance (one-way ANOVA). If the results agreed with the homogeneity of variance results, the least-significant difference (LSD) method was used for pairwise comparison, and Dunnett’s T3 method was used if the variance was uneven.

## Results

### Effect of Heshouwuyin on the MMP of spermatogenic cells

**Fig. 6:**
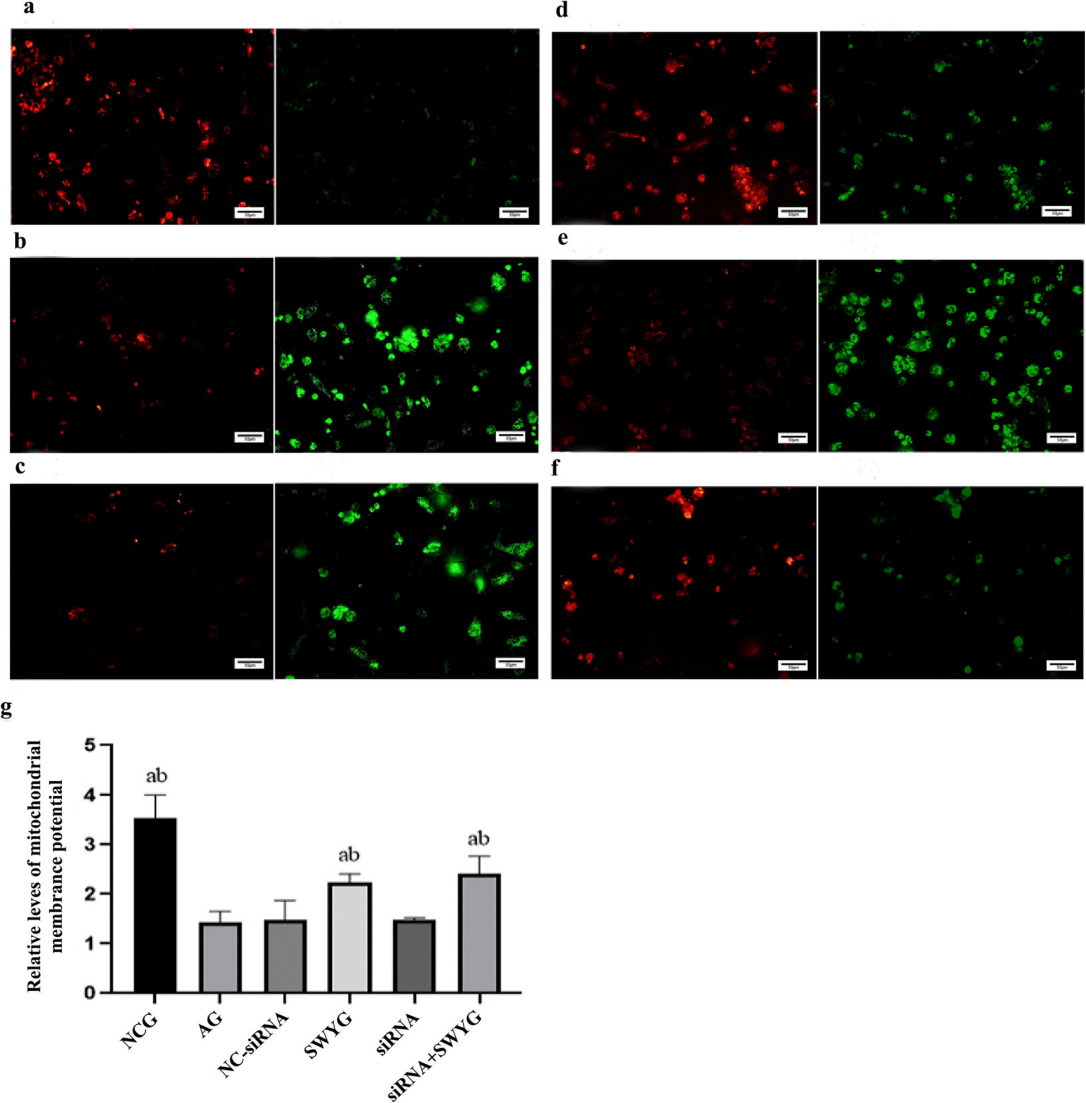
MMP changes in spermatogenic cells (bar =50 μm). **a**-**f**: Fluorescent staining showed the MMPs in spermatogenic cells (**a**: NCG; **b**: AG; **c**: NC-siRNA; **d**: SWYG; **e**: siRNA; **f**: siRNA+SWYG); **g**: Detection of the MMPs in spermatogenic cells by a microplate reader (^a^*p <* 0.05 vs. AG; ^b^*p <* 0.05 vs. Apaf-1-siRNA-transfected group) (*n* = 3)

The MMP in the aging group was lower than that in the control group, and that in the Heshouwuyin group was significantly higher than that in the aging group; all of these differences were statistically significant. This trend indicated that Heshouwuyin could stabilize the MMP. There was no significant difference in the MMP among the NC-siRNA-transfected group, the Apaf-1-siRNA-transfected group and the aging group. The MMPs in the Heshouwuyin group and co-incubation group were higher than that in the Apaf-1-siRNA-transfected group, and the difference was statistically significant. These results showed that the effect of Heshouwuyin on the MMP was dramatically stronger than the effect of Apaf-1-specific siRNA, and Apaf-1 had no effect on the MMP (Fig. 6).

### Regulatory effect of Heshouwuyin on the Cyt c, Caspase-9, and Caspase-3 proteins in spermatogenic cells

**Fig. 7:**
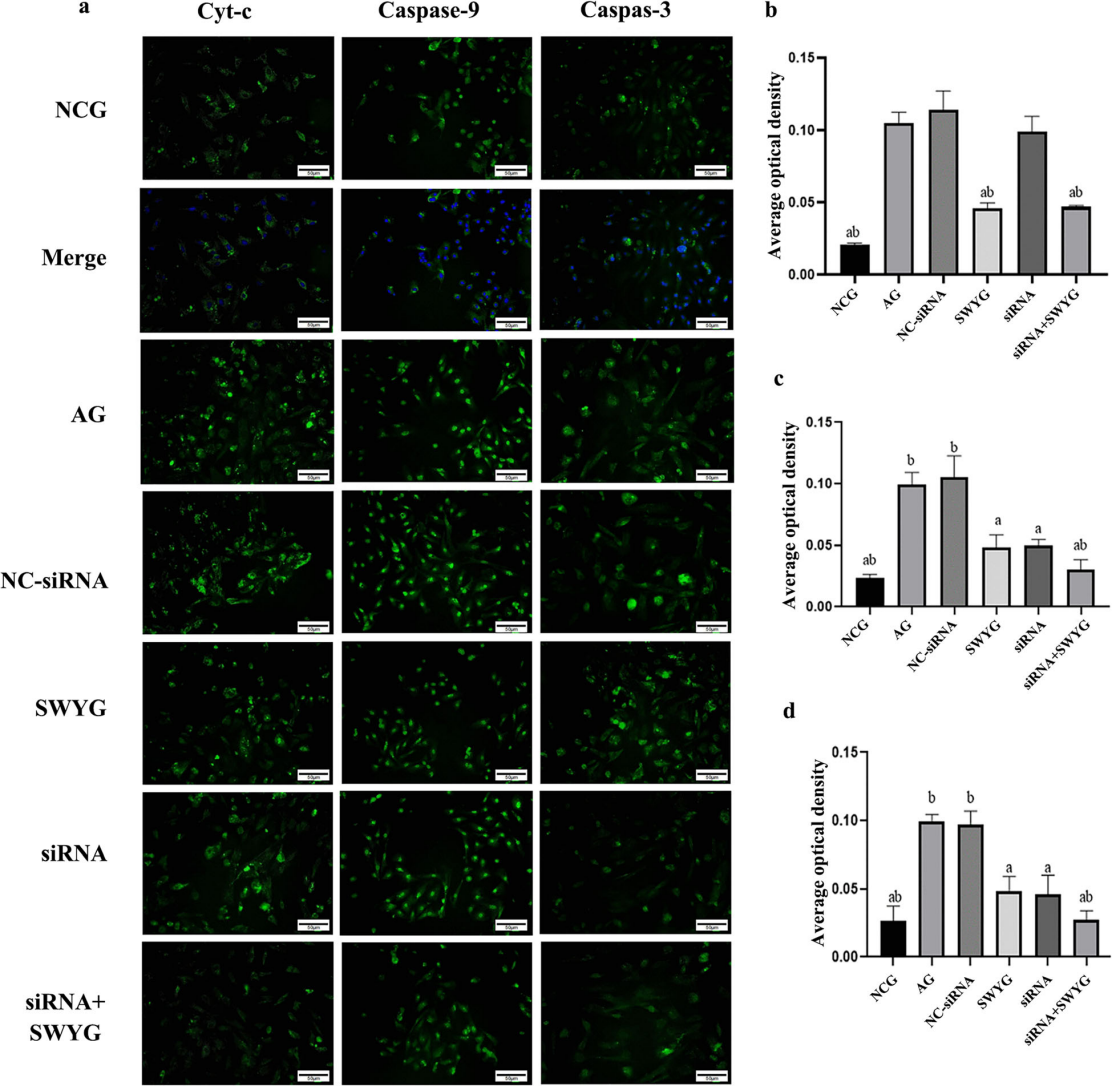
Immunofluorescence detection of the effects of Heshouwuyin on the protein expression of Cyt c, Caspase-9 and Caspase-3 in spermatogenic cells (bar = 50 μm): **a**: Immunofluorescence staining results of Cyt c, Caspase-9 and Caspase-3; **b**, **c**, **d**: Statistical analysis of average optical density of Cyt c, Caspase-9, and Caspase-3. ^*a*^*p <* 0.05 vs. AG; ^*b*^*p <* 0.05 vs. Apaf-1-siRNA-transfected group (*n* = 3)

Fluorescence immunostaining and Western blots were used for localization and semi-quantitative analysis of Cyt c, Caspase-9, and Caspase-3. The nuclear DAPI staining was blue, and the Cyt c-, Caspase-9-, and Caspase-3-positive proteins were green (Fig. 7a).

The average optical density of Cyt c, Caspase-3 and Caspase-9 immunofluorescence staining in the aging group was higher than that in the normal control group, and that in the Heshouwuyin group was significantly higher than that in the aging group. There was no significant difference between the NC-siRNA-transfected group and the aging group. The average optical density of Caspase-9 and Caspase-3 in the Apaf-1-siRNA-transfected group was lower than that in the aging group, but there was no significant difference between those in the Cyt c and aging groups. The average optical density of Cyt c, Caspase-3 and Caspase-9 in the co-incubation group was significantly lower than that in the Apaf-1-siRNA-transfected group (Fig. 7b, c, d), but there was no significant difference in the average optical density of Cyt c between the Heshouwuyin group and the co-incubation group. There was no significant difference in the average optical density of Caspase-9 and Caspase-3 between the Heshouwuyin group and the Apaf-1-siRNA-transfected group (Fig. 7b, c, d).

**Fig. 8:**
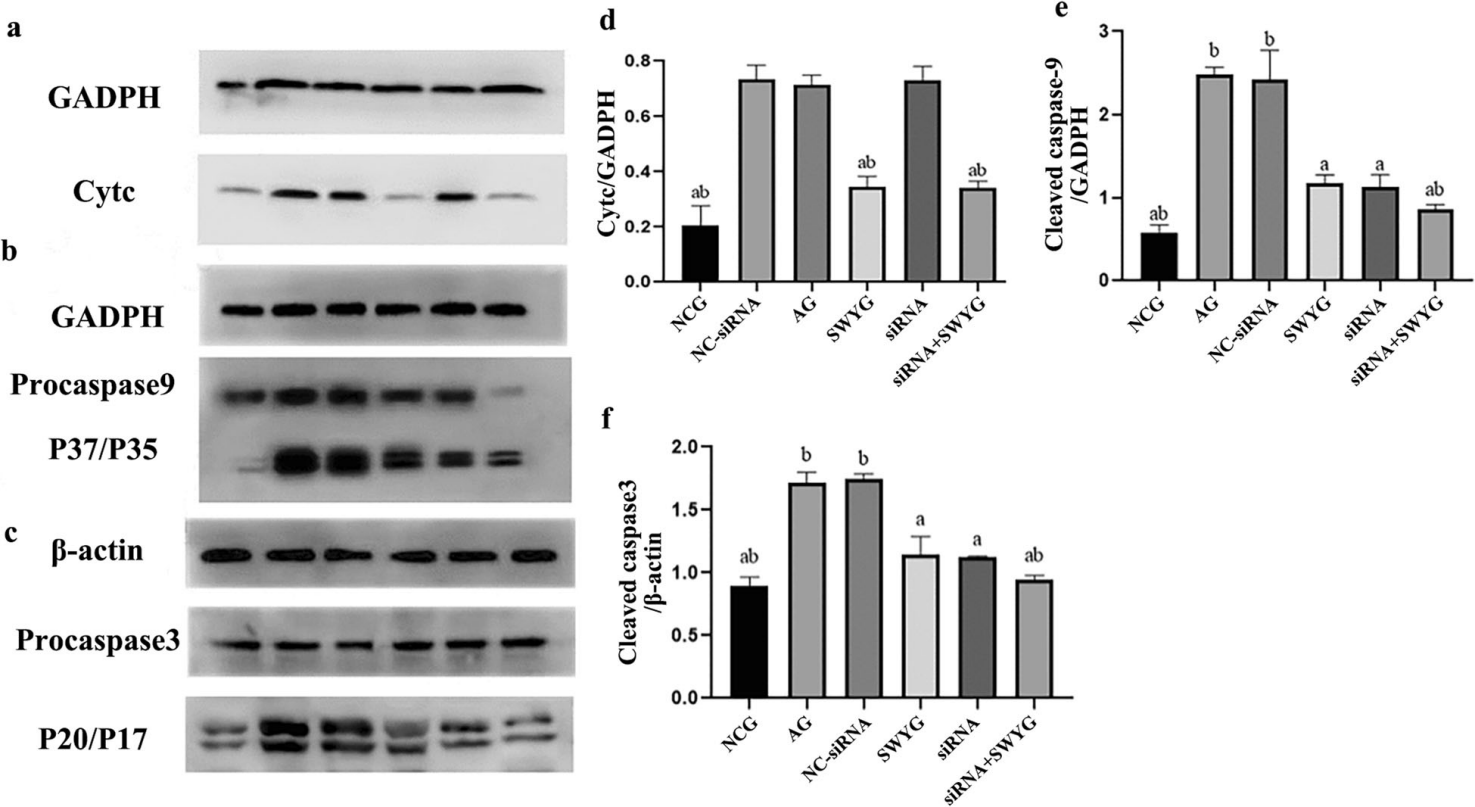
Western blot was used to detect the effects of Heshouwuyin on the protein levels of Cyt c, Caspase-9 and Caspase-3 in the spermatogenic cells of each group. **a**, **b**, **c**: Western blot results; **d**, **e**, **f**: Statistical analysis results (^*a*^*p <* 0.05 vs AG; ^*b*^*p <* 0.05 vs Apaf-1-siRNA-transfected group) 1. NCG; 2. AG; 3. NC-siRNA; 4. SWYG; 5. Apaf-1-siRNA-transfected group; 6. siRNA+SWYG (*n* = 3)

During apoptosis, apoptotic bodies recruited multiple procaspase-9 proteins and promoted their cleavage into the active subunits p37 and p35, while procaspase-3 was cleaved into the Caspase-3 p20 and Caspase-3 p17 subunits. Therefore, Western blotting was used to detect the protein levels of Cyt c, cleaved Caspase-9 and cleaved Caspase-3 in spermatogenic cells. Semi-quantitative Western blotting analysis showed that the changing protein expression trend was consistent with that seen in the immunofluorescence staining results (Fig. 8).

### Regulatory effect of Heshouwuyin on the mRNA expression of Cyt c, Caspase-9, and Caspase-3 in spermatogenic cells

**Fig. 9:**
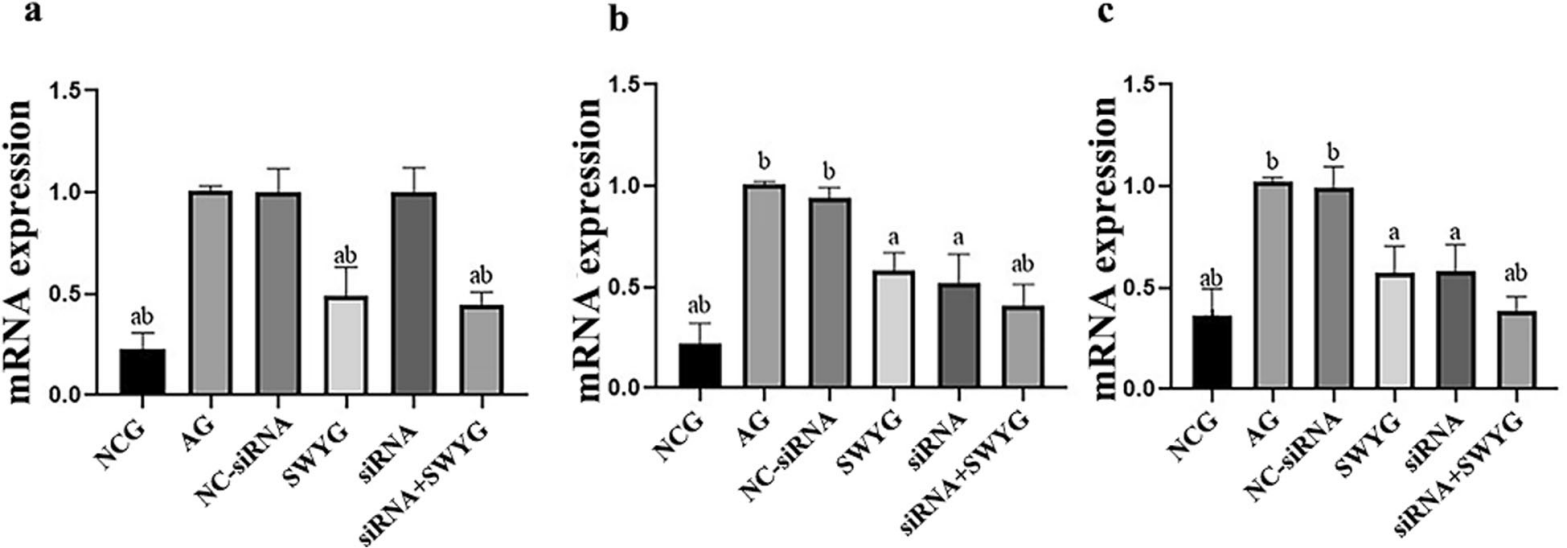
Effect of Heshouwuyin on the mRNA expression of Cyt c, Caspase-9 and Caspase-3 in spermatogenic cells. ^*a*^*p <* 0.05 vs. AG; ^*b*^*p <* 0.05 vs. Apaf-1-siRNA-transfected group (*n* = 3)

The mRNA expression level was determined by RT-qPCR. The mRNA expression levels of Cyt c, Caspase-3 and Caspase-9 were higher in the aging group than in the control group, while those in the Heshouwuyin group were lower than those in the aging group. There was no significant difference between those in the NC-siRNA-transfected group and those in the aging group. The mRNA expression levels of Caspase-9 and Caspase-3 in the Apaf-1-siRNA-transfected group were significantly lower than those in the aging group, but there was no significant difference in the Cyt c mRNA expression level between the Apaf-1-siRNA-transfected group and the aging group (Fig. 9). The mRNA expression of Cyt c, Caspase-3 and Caspase-9 in the co-incubation group was significantly lower than that in the Apaf-1-siRNA-transfected group, but there was no significant difference in the mRNA expression of Cyt c between the Heshouwuyin group and the co-incubation group. Finally, the mRNA expression of Caspase-9 and Caspase-3 between the Heshouwuyin group and the Apaf-1-siRNA-transfected group was not significantly different (Fig. 9). The results of Western blot detection were consistent with those of gene chip screening.

## Discussion

Male fertility is negatively correlated with age, and the number of germ cells and supporting cells decreases as males age [25]. Sperm production decreases and spermatogenic cell apoptosis increases, inducing male infertility (such as oligospermia, weak azoospermia and azoospermia) [26]. Reducing apoptosis is critical for spermatogenesis, but aging often breaks the balance between spermatogonia cell proliferation and apoptosis [27]. Studies have shown that the expression levels of Ki-67 (a marker of cell proliferation activity) and the apoptosis rate in the SSCs of older males are lower than those of younger control males [28]. In contrast to spermatogonia, TUNEL assays showed a significant increase in the apoptotic rate of primary spermatocytes in the older group compared with that in the younger control group, and these results partly explained the mechanism of germ cell loss in older men [28]. This difference may be due to the age-related deterioration of the SSC niche accompanied by a compensatory reduction in apoptotic spermatogonia [28]. A recent histological and ultrastructural study also showed that germ cell proliferation in senescent testis tissue decreased as apoptosis increased [29]. Apoptosis affects the development of testicular sperm, ultimately affecting male reproductive function.

Research conducted with modern technology has confirmed that Chinese medicine plays an important role in delaying aging and treating testicular dysfunction and ovarian insufficiency [30–32]. Tonifying kidney recipes (such as Cujing Decoction, Shenqi pill, Yijing Recipe, etc.) inhibit apoptosis in spermatogenic cells [33, 34]. The Chinese herbal formula Heshouwuyin was optimized from the classic clinically prescribed Heshouwu pill derived from Xuanming, which was discovered by Hejian Liu, a famous ancient Chinese physician. The Heshouwu pill mainly contains *Polygonum multiflorum* Thunb., *Cistanche deserticola*Y.C.Ma, and *Achyranthes bidentata* B1*.* and has been associated with anti-aging effects and the ability to enhance male reproductive function. Based on this, *Poria cocos* (Schw.) Wolf*, Epimedium brevicornu* Maxim. and *Salvia miltiorrhiza* Bge. were added to the ingredients of Heshouwuyin. Our previous studies have shown that Heshouwuyin enhances the activity of testosterone synthase in Leydig cells, promotes the secretion of testosterone, and improves sperm quality in aging rats [5]. Heshouwuyin can also reduce the apoptotic rate of testicular cells in natural aging rats and promote testicular cell proliferation [6]. These results confirmed that Heshouwuyin improved the spermatogenic function of aging rats, but its mechanism of action is still unclear.

SA-β-Gal is a reliable indicator to verify whether tissue or cell senescence occurs. SA-β-Gal exhibits high expression in senescent cells. In this study, a senescence model of spermatogenic cells was established by oxidative free radical damage. A recent study showed that H_2_O_2_ was used to treat oxidative damage in human retinal pigment epithelial cells (ARPE-19) [35]. Oxidative insults (FeSO_4_ and amyloid beta-peptide) induced lipid peroxidation, cellular accumulation, and apoptosis [36]. This is consistent with our findings, suggesting that the H_2_O_2_ and FeSO_4_ oxidative damage treatment method is reliable to establish a cellular senescence model. Heshouwuyin can reduce the expression of SA-β-gal in senescent spermatogenic cells, which indicates that Heshouwuyin has the effect of delaying the senescence of spermatogenic cells. However, the mechanism for delaying aging is still unknown.

It is well known that excessive apoptosis is an important factor in the development of aging and an important cause of mitochondrial dysfunction caused by aging. It can trigger mitochondrial aberration and activation of cell death pathways [37]. Apoptosis can be induced by endogenous and exogenous pathways [38]. The endogenous apoptotic pathway is a mitochondria-mediated caspase activation pathway, and our previous studies suggest that Heshouwuyin inhibition of testicular cell apoptosis may be related to the mitochondrial pathway [7]. We wondered if Heshouwuyin can inhibit spermatogenic cell apoptosis. We detected spermatogenic cells by flow cytometry and found that the apoptotic rate of senescent spermatogenic cells was significantly higher than that of normal control cells, and Heshouwuyin could inhibit senescent spermatogenic cell apoptosis. We then wondered if this inhibition of apoptosis is related to the Cyt c/Apaf-1/Caspase-3/Caspase-9 pathway.

**Fig. 10:**
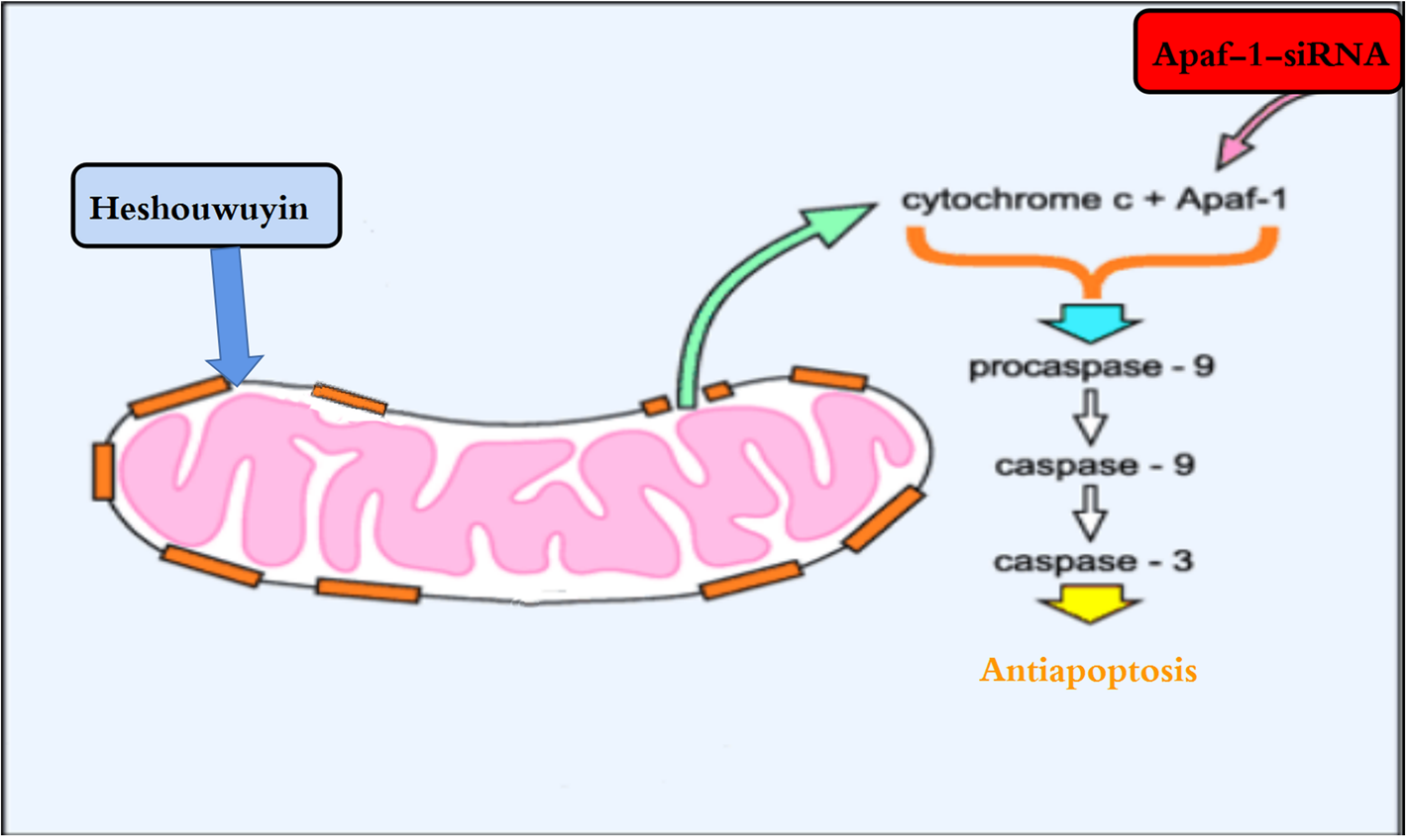
Schematic diagram of the mechanism of Heshouwuyin. Heshouwuyin reduced mitochondrial release of Cyt c by stabilizing mitochondrial membrane potential. After intervention with Apaf-1, Apaf-1 heptamer interaction with procaspase-9 was weakened, and the formation of apoptotic bodies was reduced, procaspase-9 was cleaved to suppress the expression of Caspase-9 and Caspase-3, thereby inhibiting the apoptosis of spermatogenic cells. However, it was also shown in the results that the effect of Heshouwuyin on the inhibition of spermatogenic cell apoptosis was also regulated by other signaling pathways

RNA interference technology is widely used in functional analysis experiments. siRNA can efficiently block the expression of specific genes by degrading mRNA to make cells express a phenotype of specific gene deletions [39]. Apoptotic protease activating factor-1 (Apaf-1), a major component of the apoptotic complex, is a key factor in the mitochondria-dependent apoptotic pathway. When the endogenous apoptotic pathway is activated in human or murine cells, apoptotic stimuli can induce mitochondrial outer membrane permeabilization (MOMP) through the Bcl-2 protein family, promoting the release of Cyt c from mitochondria. This step is considered to be a key signal for an irreversible event that triggers apoptosis [40]. Studies have shown that Heshouwuyin can increase the expression of the Bcl-2 protein and can decrease the expression of BAX in aging rats [7]. This study found that Heshouwuyin can stabilize the MMP of spermatogenic cells and reduce the mRNA and protein expression levels of Cyt c, Caspase-3 and Caspase-9 in senescent spermatogenic cells. Therefore, it is speculated that Heshouwuyin can reduce the release of Cyt c in mitochondria and decrease the formation of the Apaf-1 heptamer by stabilizing the MMP, thereby inhibiting the apoptosis of spermatogenic cells. Studies have shown that in the presence of Cyt c and deoxyadenosine triphosphate (dATP), ATP binds to Apaf-1 to oligomerize it into the Apaf-1 heptamer [41]. The CARD domain exposed in the Apaf-1 heptamer interacts with the initiator procaspase-9 to form an apoptotic somatic enzyme [42]. Once an apoptotic body is formed, procaspase-9 is cleaved and activated to further activate an executioner caspase, such as Caspase-3. Activation of Caspase-3 results in DNA fragmentation and PARP cleavage, which in turn triggers a caspase-dependent apoptotic signalling cascade [43]. Therefore, in this study, Apaf-1 siRNA was used to silence the Apaf-1 gene in spermatogenic cells and to explore the mechanism by which Heshouwuyin inhibits spermatogenic cell apoptosis through the Cyt c/Apaf-1/Caspase-3/Caspase-9 pathway (Fig. 10).

This study found that after Apaf-1-siRNA interference, the mRNA and protein expression levels of Caspase-3 and Caspase-9 in the spermatogenic cells of the control group were significantly lower than those of the aging group, while the mRNA and protein expression levels of Cyt c were not statistically significant in the aging group. This result demonstrated that knockdown of Apaf-1 reduced the mRNA and protein expression levels of the downstream proapoptotic genes Caspase-9 and Caspase-3 and inhibited apoptosis of spermatogenic cells, while Cyt c was the upstream gene of Apaf-1. Finally, Apaf-1 knockdown had no significant effect on Cyt c expression. In this study, the Apaf-1 gene was specifically knocked down in the spermatogenic cells, and serum containing Heshouwuyin was used to treat spermatogenic cells. The results showed that the expression levels of the proapoptotic factors Caspase-9 and Caspase-3 in the SWYG+Apaf-1-siRNA group were lower than those in the Heshouwuyin group and the Apaf-1-siRNA-transfected group, indicating that the inhibition of spermatogenic cell apoptosis by Heshouwuyin is closely related to the Cyt c/Apaf-1/Caspase-9/Caspase-3 pathway. However, the inhibition of apoptosis by Heshouwuyin was significantly stronger than the inhibition by Apaf-1-specific siRNA, indicating that the effect of Heshouwuyin inhibiting spermatogenic cell apoptosis was also regulated by other signalling pathways.

## Conclusion

The inhibition of spermatogenic cell apoptosis by Heshouwuyin was closely related to the Cyt c/Apaf-1/Caspase-9/Caspase-3 pathway. The inhibition of apoptosis by Heshouwuyin involved not only the Apaf-1 pathway but also other signaling pathways.

## Data Availability

All data used and analyzed during the current study available from the corresponding author on reasonable request.

## Abbreviations

Apaf-1: Apoptotic protease activating factor-1
Cytc: Cytochrome c
Caspase-9: Cysteinyl aspartate specific proteinas 9
Caspase-3: Cysteinyl aspartate specific proteinas 3
SSCs: Spermatogonial stem cells
FBS: Fatal Bovine Serun
DMEM/F12: Dulbecco’s Modified Eagle Medium/Nutrient Mixture F-12
MMP: Mitochondrial membrance potential
DAPI: 4′,6-diamidino-2-phenylindole
RIPA: Radio-Immunoprecipitation Assay
PMSF: Phenylmethanesulfonyl fluoride
PI: Propidiumiodile
PBS: Phosphate buffered saLine
SA-β-gal: Senescence-associated β-galactosidase
NCG: Normal control group
AG: Aging group
NC-siRNA: Normal control siRNA-transfected group
SWYG: Heshouwuyin group
Apaf-1-siRNA: Apaf-1-siRNA-transfected group
Apaf-1-siRNA +SWYG: Apaf-1-siRNA + Heshouwuyin group
